# TiO_2_@C core-shell nanoparticles formed by polymeric nano-encapsulation

**DOI:** 10.3389/fchem.2014.00047

**Published:** 2014-07-11

**Authors:** Mitra Vasei, Paramita Das, Hayet Cherfouth, Benoît Marsan, Jerome P. Claverie

**Affiliations:** Department of Chemistry, NanoQAM, Québec Center for Functional Materials, Université du Québec à MontréalMontreal, QC, Canada

**Keywords:** RAFT polymerization, encapsulation, TiO_2_, photocatalysis, carbon, polyacrylonitrile

## Abstract

TiO_2_ semiconducting nanoparticles are known to be photocatalysts of moderate activity due to their high band-gap and high rate of electron-hole recombination. The formation of a shell of carbon around the core of TiO_2_, i.e., the formation of TiO_2_@C nanoparticles, is believed to partly alleviate these problems. It is usually achieved by a hydrothermal treatment in a presence of a sugar derivative. We present here a novel method for the formation of highly uniform C shell around TiO_2_ nanoparticles. For this purpose, TiO_2_ nanoparticles were dispersed in water using an oligomeric dispersant prepared by Reversible Addition-Fragmentation chain Transfer (RAFT) polymerization. Then the nanoparticles were engaged into an emulsion polymerization of acrylonitrile, resulting in the formation of a shell of polyacrylonitrile (PAN) around each TiO_2_ nanoparticles. Upon pyrolysis, the PAN was transformed into carbon, resulting in the formation of TiO_2_@C nanoparticles. The structure of the resulting particles was elucidated by X-Ray diffraction, FTIR, UV-VIS and Raman spectroscopy as well as TEM microscopy. Preliminary results about the use of the TiO_2_@C particles as photocatalysts for the splitting of water are presented. They indicate that the presence of the C shell is responsible for a significant enhancement of the photocurrent.

## Introduction

Since the landmark report on the photoelectrochemical splitting of water by TiO_2_ (Fujishima and Honda, 1972) TiO_2_ nanoparticles have attracted much attention,(Linsebigler et al., 1995) and found applications in dye-sensitized photovoltaic devices (O'Regan and Grätzel, 1991; Grätzel, 2009) for the photodegradation of organic pollutants (Wold, 1993; Hoffmann et al., 1995) and for the production of hydrogen (Ni et al., 2007). TiO_2_ is a choice material for these usages because of its high stability, low cost and low toxicity. However, due to its high band gap energy (3.2 eV), TiO_2_ (anatase) is only activated by UV-light, which merely constitutes 5% of solar spectrum. The extension of the TiO_2_ active window to the visible portion of sunlight spectrum is the object of considerable scrutiny. For example, TiO_2_ can be doped with nitrogen (Burda et al., 2003), carbon (Park et al., 2006), boron (Chen et al., 2006), or sulfur (Tang and Li, 2008) in order to achieve this goal. However, the dopant tends to distort the TiO_2_ lattice, resulting in an increase in the rate of electron/hole recombination. As an alternative, TiO_2_- C composites have been investigated (Inagaki et al., 2005; Shanmugam et al., 2006; Zhang et al., 2010; Zhao et al., 2010; Jang et al., 2012; Olurode et al., 2012; Zheng et al., 2013), because the presence of carbon is beneficial to transport the charges. In these materials, it is believed that electron hole pairs are photogenerated in the TiO_2_, but rapidly recombine due to the presence of defects in the crystalline structure. With its zero-band gap, the carbon layer acts as a sink for the electrons. Thus electron-hole recombination is attenuated due to a better charge separation, and the electrons are channeled from the semi-conductor to the device electrodes, thus permitting the flow of the electrical current. Furthermore, due to the coupling of π states of the graphitic material with the conduction band of TiO_2_, the activity window is extended to the visible spectrum.

The deposition of a carbon layer on TiO_2_ nanoparticles is usually achieved via a hydrothermal process, whereby the particles are coated with an organic precursor which is then thermally decomposed to yield a carbon layer. For example, glucose (Zhang et al., 2011; Zheng et al., 2013), dextrose (Olurode et al., 2012), carboxy methyl cellulose (Inagaki et al., 2005) and polyvinyl alcohol (Tsumura et al., 2002) have been used in the past as precursors to coat TiO_2_ nanoparticles. However, this process does not usually allow control over the carbon layer thickness and uniformity, resulting in products which are opaque to light. Thus, the advantages bestowed by the presence of a carbon layer (larger surface area, easier charge transport, reduced recombination rate) can be offset by the reduction of UV intensity actually reaching the TiO_2_ surface.

**Figure 1:**
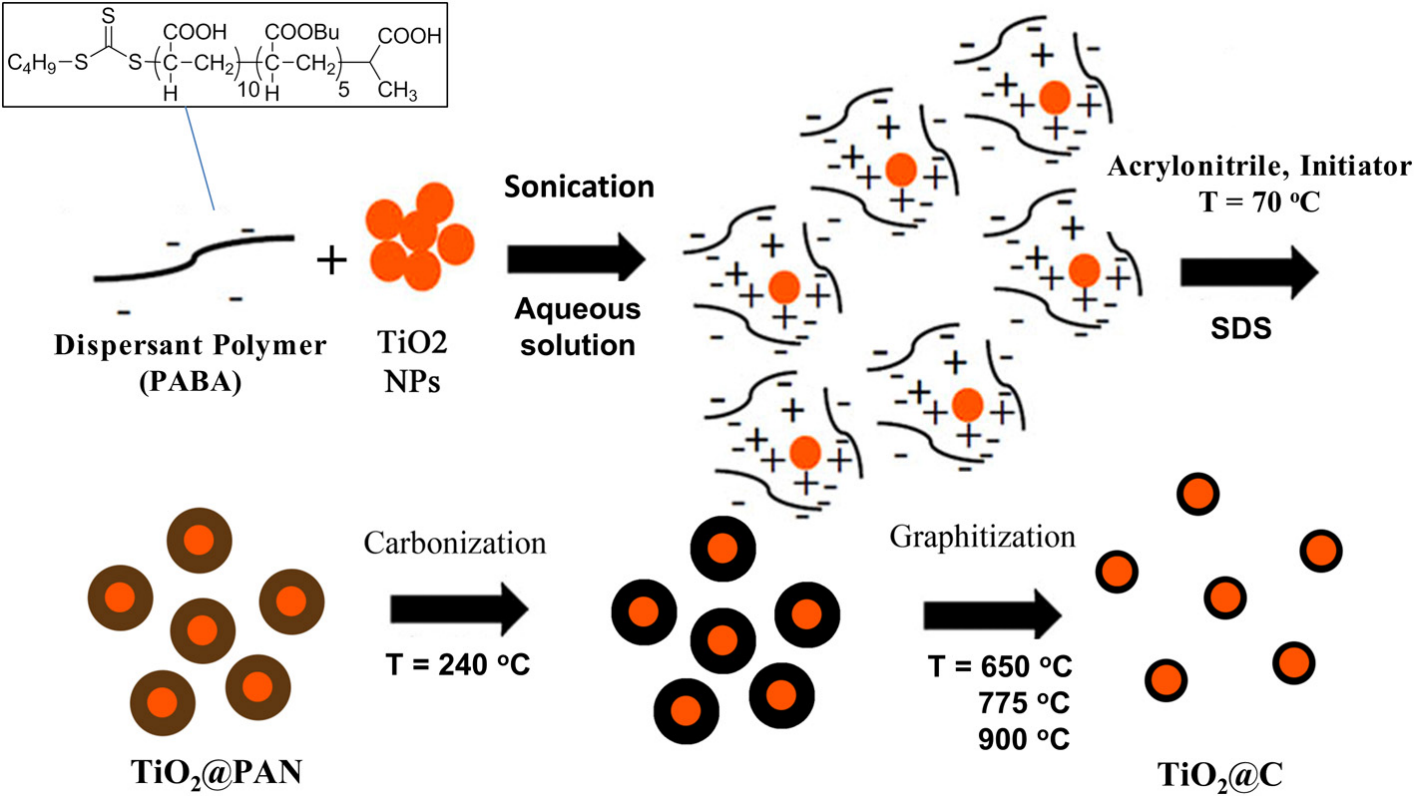
**Process for the formation of TiO_2_@C nanoparticles**. The first step is the dispersion of agglomerated TiO_2_ nanoparticles in water with a small amount of PABA dispersant. Electrostatic interaction occurs between positively charged TiO_2_ surface and negatively charged PABA. The second step is an emulsion polymerization process resulting in the formation of a layer of polyacrylonitrile. The third or fourth steps are respectively carbonization and graphitization steps, whereby PAN is converted in graphite.

In this work, we propose a novel templating process to prepare TiO_2_@C nanoparticles, that is to say core-shell particles where the core is TiO_2_ and the shell graphitic carbon. For this purpose, we have exploited the ability of polyacrylonitrile, PAN, to convert cleanly into graphite when heated under controlled atmospheric conditions. Thus, highly uniform TiO_2_@PAN nanoparticles were prepared, using an *in-situ* nanoencapsulation method that we recently devised (Das et al., 2011; Das and Claverie, 2012; Zhong et al., 2012) to encapsulate quantum dots and carbon nanotubes (Figure 1). This method is based on the use of a so-called controlled polymerization technique, Reversible Addition-Fragmentation chain Transfer (RAFT) polymerization. After carbonization, the TiO_2_@C nanoparticles were obtained with a highly uniform shell of carbon. Advantageously, the thickness of the shell could be tailored by simply changing the amount of PAN. To our knowledge, the use of RAFT polymerization technique for the encapsulation is the only method which allows forming 1 to 1 core shell nanoparticles, whereby each TiO_2_ nanoparticle is surrounded by a continuous and size-tunable shell of PAN, and no free PAN or TiO_2_ nanoparticles are observed. This paper concentrates on the fabrication and characterization of these novel hybrid nanoparticles, and their conversion into TiO_2_@C, upon pyrolysis. Preliminary results on the photocatalytic activity of TiO_2_@C particles for the water splitting reaction are also presented.

## Materials and methods

### Materials

All chemicals were purchased from Sigma-Aldrich and used without further purification unless specifically mentioned. Indium tin oxide (ITO) glass was graciously offered by the Pilkington company. Just prior use, acrylonitrile (AN) was passed over a column of basic alumina in order to remove the polymerization inhibitor. The random copolymer PABA, containing an average of five butyl acrylate and 10 acrylic acid units was prepared according to literature (Nguyen et al., 2008; Ali et al., 2009). Outmost care should be used to manipulate acrylonitrile which is highly toxic. For this work, three different kinds of TiO_2_ were used. Rutile nanoparticles of average diameter 20 nm (specific surface 30 m^2^/g), anatase nanoparticles of average diameter 20 nm (specific surface 200–220 m^2^/g) and P25 nanoparticles from Evonik of average size 25 nm (80:20 mixture of anatase and rutile, specific surface 30–65 m^2^/g).

### Preparation of the TiO_2_@C nanoparticles and electrodes

#### Preparation of TiO_2_@PAN

Concentrated NaOH (19.4 mol/L) was added dropwise to a suspension of PABA (12.5 g/L) in water until pH increased to 6, point at which the PABA was fully dissolved (slight heating was necessary). Dry TiO_2_ nanoparticles (2g) were added to 20 mL of PABA solution and stirred for half an hour. Then, the suspension was sonicated with a Fisher scientific sonic dismembrator model 500 for 15 min at 30% amplitude (400 W). During the sonication, the suspension was maintained at room temperature using a cooling bath and was also magnetically stirred, and care was taken in order the magnetic stir bar not to touch the sonicating probe. A colloidaly stable dispersion of TiO_2_ in water was thus obtained. To this dispersion, 50 mg of sodium dodecyl sulfate (SDS) and AN (amount as listed in Table 1) were added, and was degased by bubbling nitrogen for 20 min. The emulsion was then stirred and heated at 70°C under a continuous blanket of nitrogen, and 20 mL of a degassed solution of 4,4 azobis (4 cyanovaleric acid), ABVA, (*c* = 8.9 mmol/L) was added over 4 h (5 ml/h rate) under nitrogen atmosphere. Then, the reaction was stirred for an additional 2 h at 70°C in order to complete the polymerization, yielding a white colloidaly stable dispersion of TiO_2_@PAN nanoparticles.

**Table 1:** **Composition of encapsulated TiO_2_ nanoparticles**.

**Sample**	**TiO_2_**	**TiO_2_ (g)**	**PABA (g)**	**AN (g)**	**ABVA (g)**	**SDS (g)**
R1	Rutile	2.0	0.25	2.0	0.05	0.05
P1	P25	2.0	0.25	2.0	0.05	0.05
A1	Anatase	2.0	0.25	2.0	0.05	0.05
A2	Anatase	2.0	0.25	4.0	0.05	0.05
A3	Anatase	2.0	0.25	1.0	0.05	0.05

#### Preparation of TiO_2_@C

In a Petri dish, 1 mL of the TiO_2_@PAN dispersion was dried in air, to yield a white powder which was transferred to a ceramic crucible. In an oven, the powder was heated under air to 240°C at a rate of 11°C/min. This carbonization step promotes the intramolecular cyclization of PAN, to yield a conjugated ladder structure which acts as a precursor for graphite-like materials. During this carbonization step, release of hydrocyanic (HCN) acid and organic nitriles occurs (Usami et al., 1990), and suitable measures were taken to trap them and ensure safety of the experimentator. Once the temperature of 240°C reached, the oven was switched to nitrogen, and heated at a rate of 5°C/min. Three final temperatures were tested: 650, 775, and 900°C (Figure 1).

#### Preparation of TiO_2_@C electrode

To analyze the electrochemical behavior of the TiO_2_@C nanoparticles, the powder samples were applied on ITO coated glass plates. However, it was soon discovered that due to high surface tension of ITO, the TiO_2_@C powder did not adhere to the ITO surface when immersed in water. Thus, in order to render ITO surface more hydrophobic and to obtain a uniform and continuous coating of TiO_2_@C, the ITO surface was first treated with n-octyltrichlorosilane in a n-heptane: tetrachloromethane (7:3 v:v) solution. The detailed procedure for this silanization treatment can be found in (Choi et al., 2001). In 150 μL of N-methyl-pyrrolidone (NMP), polyvinylidene difluoride (PVDF, 0.3% (w/v) in NMP) and TiO_2_@C powder (4.85 mg) were added. The resulting suspension was sonicated for 5 min, using the same conditions as described in Preparation of TiO_2_@PAN. The resulting dispersion was then dripped on silanized ITO and spread to form a uniform film, and the resulting film was placed in a vacuum-oven at 40°C for 48 h.

### Characterizations

#### Fourier-transform infrared (FTIR) spectroscopy

Dry samples were analyzed on a Nicolet 6700 Spectrometer equipped with Smart ATR accessory (ThermoSci).

#### RAMAN spectroscopy

In order to assess the nature of the carbon layer, TiO_2_@C nanoparticles were characterized by Raman spectroscopy, using a Renishaw RM3000 RAMN microscope equipped with 514.5 nm excitation laser and a CCD detector. The RAMAN signals were acquired in a backscattering geometry with a 50× magnification.

#### Transmission electron microscopy (TEM)

TEM analysis was performed on carbon coated copper grids (mesh 200) using a JEOL JEM-2100F microscope operating at 200 kV acceleration voltage. Samples were diluted with nanopure water in order to reach an approximate concentration of 0.05 wt% in water. For TiO_2_@C nanoparticles, the dispersion was sonicated after dilution in order to prevent the formation of aggregates (this step was not necessary for TiO_2_@PAN nanoparticles as they were stabilized by surfactant). A 10 μL drop was deposited on each grid, and left to dry in air for 12 h. A large number of TEM pictures are presented as supplementary material.

#### X-ray diffraction (XRD)

All samples were analyzed by XRD in order to assess possible structural changes to TiO_2_ crystalline structure, using a Panalytical X'Pert diffractometer with Cu K_α_ source at 1.5405 Å, operating with a maximum voltage of 50 kV and current of 40 mA, and by varying 2θ between 20 and 80°.

### Electrochemical characterization

#### Cyclic voltammetry (CV)

CV experiments were performed at room temperature in a one-compartment 125 mL glass cell using a three-electrode configuration, a TiO_2_@C electrode of surface 0.25 cm^2^ as working electrode, a counter electrode of platinum and a reference electrode of calomel. The electrolyte was an aqueous solution of Na_2_SO_4_ (0.5 mol/L) acidified with sulfuric acid to pH = 2.5. Voltage was swept from 0.5 to 1.4 V at scan rate varied between 25 and 200 mV/s. Several CVs can be found in supplementary material section.

#### Electrochemical impedance spectroscopy (EIS)

EIS experiments were performed at room temperature in a custom-made one compartment 125 mL glass cell fitted with a large opening on its top in order light not to be blocked. A three electrode configuration was used, using, a TiO_2_@C electrode of surface 1 cm^2^ as work electrode, a counter electrode of platinum and a reference electrode of Ag/AgCl (0.242 V vs. ENH). The electrolyte was an aqueous solution of Na_2_SO_4_ (0.5 mol/L) acidified with sulfuric acid to pH = 2.5. The electrolyte was purged with argon before the experiment. The signals were generated with a Solartron 2255B with an AC perturbation signal of 10 mV, and frequencies varied between 100 mHz and 1 MHz. The data points were recorded and analyzed with the help of the Zplot/ZView software. The EIS experiments were performed in darkness, under solar illumination provided by a solar simulator SS500W from Sciencetech or under UV illumination provided by a 500 W high-pressure Xenon lamp. The distance from the mirror to the electrode was 20 cm. The nominal light intensity was 80 mW/cm^2^, and the recorded intensity at the electrode surface was 3 mW/cm^2^ for the UV lamp and 3.7 mW/cm^2^ for the solar simulator.

#### Potentiostatic measurements

Photocurrents were measured in experiments were performed at room temperature in a custom-made one compartment 125 mL glass cell fitted with a large opening on its top in order light not to be blocked. A three electrode configuration was used, using, a TiO_2_@C electrode of surface 1 cm^2^ as work electrode, a counter electrode of platinum and a reference electrode of Ag/AgCl. The electrolyte was an aqueous solution of Na_2_SO_4_ (0.5 mol/L) acidified with sulfuric acid to pH = 2.5. The electrolyte was purged with argon before the experiment. The voltage was maintained at 1.5 V, and the current was recorded while alternating 1 min cycles of light and darkness. Two different light sources were used: solar illumination provided by a solar simulator SS500W from Sciencetech or UV illumination provided by a 500 W high-pressure Xenon lamp. The distance from the conducting mirror to the electrode was 20 cm. The nominal light intensity in both cases was 80 mW/cm^2^, and the recorded intensity at the electrode surface was 3 mW/cm^2^ for the UV lamp and 3.7 mW/cm^2^ for the solar simulator. Several potentiostatic curves can be found in Supplementary Material section.

## Results

### Preparation of TiO_2_@PAN nanoparticles

The first step for the preparation of the core-shell nanoparticles consists in the preparation of a colloidaly stable dispersion of TiO_2_ nanoparticles in water, using a polymeric dispersant prepared by RAFT polymerization. In this work we used the RAFT dispersant PABA which is a random copolymer of acrylic acids (10 units) and butyl acrylate (5 units), which was first reported by Ferguson et al. (2005) and used at several occasions either by the Hawkett group (Nguyen et al., 2008, 2013; Ali et al., 2009) or by our group (Das et al., 2011; Das and Claverie, 2012; Zhong et al., 2012). Other polymeric dispersants were also assessed, such as random copolymers of styrene and acrylic acid (Zhong et al., 2012) or homopolymers of acrylic acid (Daigle and Claverie, 2008), but they were found to be less efficient during the subsequent encapsulation, and they were not further investigated. Dispersions prepared with 2.0 g of TiO_2_ (rutile, anatase or P25) and 0.25 g of PABA in 20 mL of water were found to be colloidaly stable over several days, as shown by the absence of visual aggregates. In past studies (Daigle and Claverie, 2008; Zhong et al., 2012), we have shown that PABA is adsorbed at the surface of the nanoparticle owing to a combination of hydrophobic interactions between the butyl acrylate and the surface and electrostatic interactions between negatively charged acrylic acids and positively charged surface patches.

**Figure 2:**
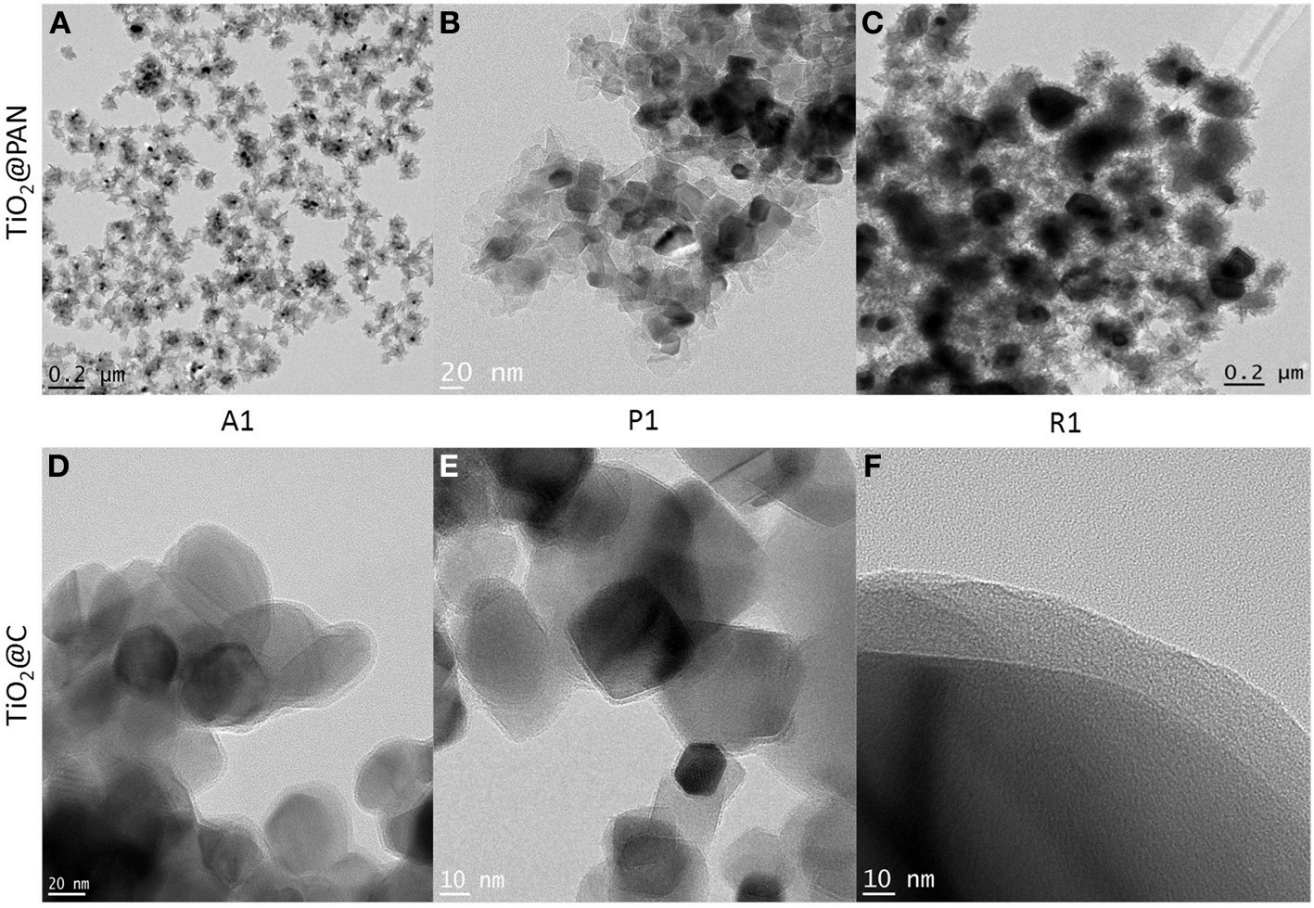
**TEM pictures of TiO_2_@PAN nanoparticles (A–C) and TiO_2_@C nanoparticles (D–F)**. Anatase: **(A,D)**, P25: **(B,E)**, rutile **(C,F)**. The corresponding sample composition (A1, P1 or R1) is indicated in Table 1 and the carbon layer thickness in Table 2. In **(D,E)**, several TiO_2_@C nanoparticles are superimposed (as shown by darker contrast).

The TiO_2_ dispersion in water was then engaged in an emulsion polymerization of AN. If the TiO_2_ nanoparticles were stabilized by a conventional surfactant, the emulsion polymerization process would lead to the formation of a PAN latex, that is to say separated PAN nanoparticles and TiO_2_ nanoparticles. However, in our case the dispersant has been prepared by RAFT polymerization which is a controlled radical polymerization process. Therefore, when AN is consumed, a block copolymer is formed whereby the first block is PABA and the second is a growing chain of PAN. Thus, PAN is covalently anchored to the PABA dispersant with itself is located at the TiO_2_ surface. As a large excess of AN is used compared to PABA (Table 1), the TiO_2_ is effectively engulfed within PAN and core-shell TiO_2_@PAN nanoparticles are thus formed. In Figure 2, the nanoparticles are characterized by TEM, demonstrating that neither free TiO_2_ particles nor free PAN particles are present in the sample. The flaky aspect of the polymer is due to the high glass transition temperature, Tg, of PAN (95°C) as well as its native crystallinity (Hobson and Windle, 1993) which prevents the polymer chains from adopting a spherical shape. Interestingly, the sample is colloidaly stable after the emulsion polymerization process, and it is devoid of aggregates. Thus, the use of the RAFT polymerization process is an efficient and simple method to form TiO_2_@PAN nanoparticles. It should be mentioned that no attempt was made to check whether the polymerization of AN was actually well-controlled (for example by isolating the polymer and by checking its polydispersity), as the subsequent step consists in its pyrolysis.

### Preparation and characterization of TiO_2_@C nanoparticles

The formation of graphitic carbon upon pyrolysis of organic molecules, and most notably PAN, is by now a well-established technique (Zussman et al., 2005; Ismail and Li, 2008; Kong et al., 2010; Thomassin et al., 2010). The layer of carbon is obtained by a two-step process (Figure 1). First a carbonization or stabilization process, performed in air by heating the TiO_2_@PAN nanoparticles up to 240°C, leads to the formation of conjugated ladder-like structures. Then, this carbonaceous material is heated under nitrogen to three different temperatures 650, 775, and 900°C, in order to investigate the effect of the final graphitization temperature on the structure of the hybrid nanoparticle.

**Figure 3:**
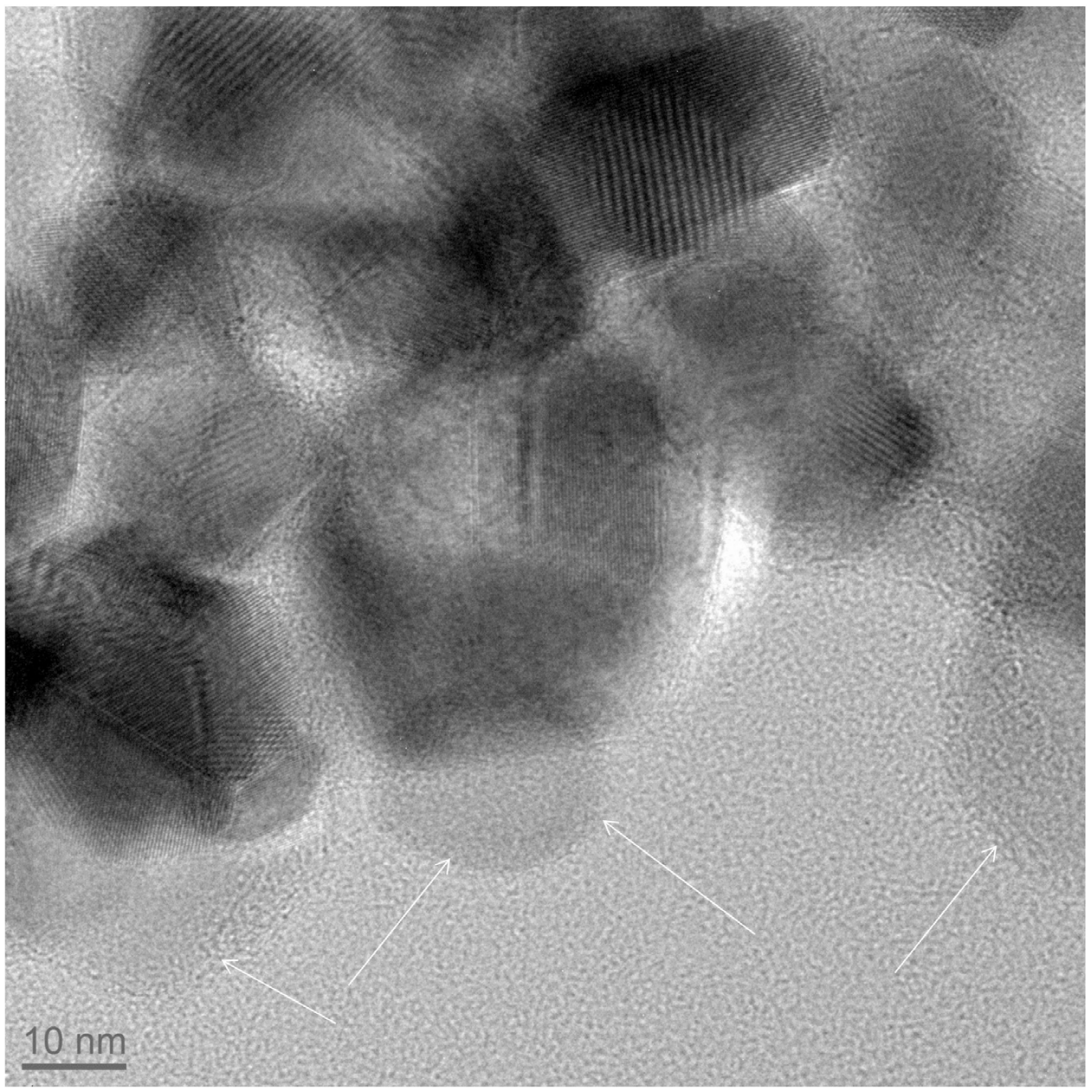
**TiO_2_@C (TiO_2_ = anatase) nanoparticles prepared by a conventional hydrothermal synthesis, following the procedure of (Olurode et al., 2012)**. The arrows outline carbon particles.

The formation of a carbon shell was evident in TEM pictures (Figures 2D–F), where TiO_2_ and C were identified by electron diffraction (the carbon layer did not diffract) and by energy dispersive X-ray (EDX) analysis (Supplementary material). The carbon shell is also remarkably uniform and continuous: no large carbon aggregates or carbon nanoparticles could be observed by TEM. The thickness of the carbon shell was measured on TEM pictures at more than 50 different locations in order to obtain a statistical sampling (Table 2). For the sake of comparison, TiO_2_@C nanoparticles were prepared using the conventional hydrothermal process, whereby the TiO_2_ nanoparticles are first impregnated with dextrose, and the sugar is then decomposed under heat and pressure (Figure 3).

**Table 2:** **Degree of graphitization (D/G) obtained from RAMAN spectroscopy, band gap energy measured by Tauc's plot (Tauc et al., 1966), thickness and standard deviation *SD* of the C shell as measured by TEM microscopy**.

	**Degree of graphitization**			**Band gap (eV)**	**C Thickness (nm)**	
	**650°C**	**775°C**	**900°C**		**Average (nm)**	***SD* (%)**
R1	1.21	nd	nd	2.9	15.8	31
rutile@C	nd	nd	nd	nd	3.2	83
P1	1.28	nd	nd	2.7	1.4	24
P25@C	nd	nd	nd	nd	3.1	79
A1	1.31	0.96	0.93	2.6	8.9	26
A2	1.33	1.09	0.83	2.2	12.4	31
A3	1.30	0.85	0.86	2.5	5.1	21
anatase@C	nd	nd	nd	nd	2.2	146

*Bandgap and C thickness were determined for samples graphitized at 650 °C. The samples rutile@C, P25@C and anatase@C are control experiments performed via hydrothermal treatment of dextrose (Olurode et al., 2012)*.

**Figure 4:**
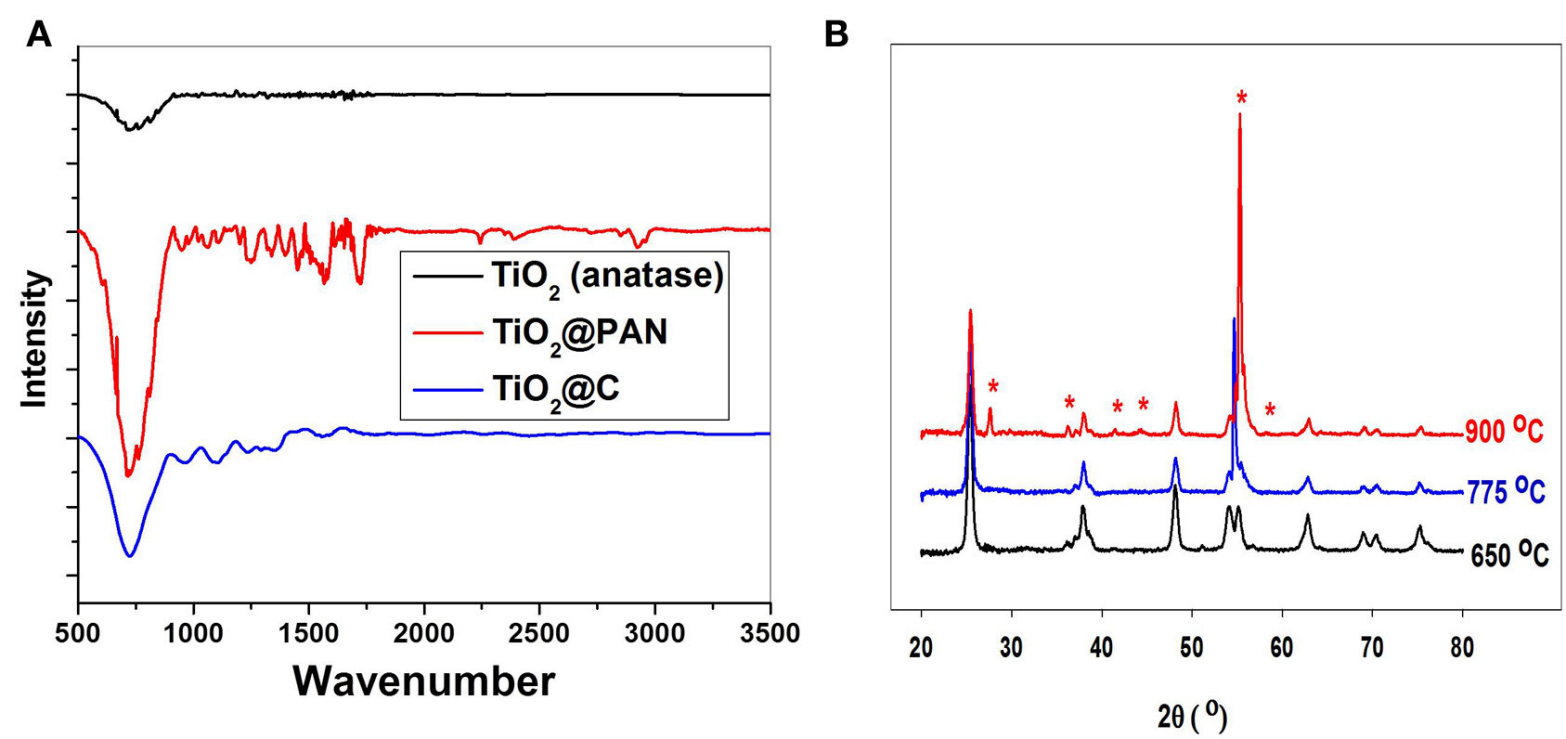
**(A)** FTIR of anatase nanoparticles A1, before encapsulation, after polymer encapsulation and after carbonization at 775°C. **(B)** X-ray diffraction of TiO_2_@C (sample A2) after carbonization at three different temperatures. The peaks marked with a star correspond to rutile crystallites.

Evidence for the clean conversion of PAN into carbon is brought by FTIR spectroscopy. The band near 700 cm^−1^ corresponds to the Ti-O stretching vibration, and is present in TiO_2_, TiO_2_@PAN and TiO_2_@C samples (Figure 4A). However, the band at 2243 cm^−1^, characteristic of CN stretching, is only present in TiO_2_@PAN and not in TiO_2_ and TiO_2_@C, indicating that the nitrile functionality completely disappears during the pyrolysis process.

Importantly, the pyrolysis process did not affect the structure of TiO_2_ provided the temperature was kept below 775°C. It is well known that at higher temperatures, anatase is converted in rutile, and indeed, the sample which was pyrolyzed up to 900°C contained a significant amount of rutile, as shown by XRD analysis (Figure 4B). By TEM, spherical anatase particles are observed for samples pyrolyzed at 650 and 775°C, but samples pyrolyzed at 900°C exhibit a rod-like structure characteristic of rutile (see supplementary material). The anatase to rutile transition is known to occur at around 600°C for pure TiO_2_, but for carbon covered TiO_2_ nanoparticles this temperature is shifted to higher temperatures due to the shielding effect of the graphite coating (Inagaki et al., 2005). Excepted for the transition to rutile at 900°C, the shape and size of the TiO_2_@C nanoparticles is conserved during the graphitization process. Thus, for sample A2, the particle size as measured by TEM is respectively 17, 19, and 25 nm for the sample graphitized at 650, 775, and 900°C. The nanoparticle size is also in good agreement with the crystallite size measured by XRD using the Debye-Scherrer equation, which was respectively 14.7, 16.0, and 17.0 nm for the sample graphitized at 650, 775, and 900°C.

**Figure 5:**
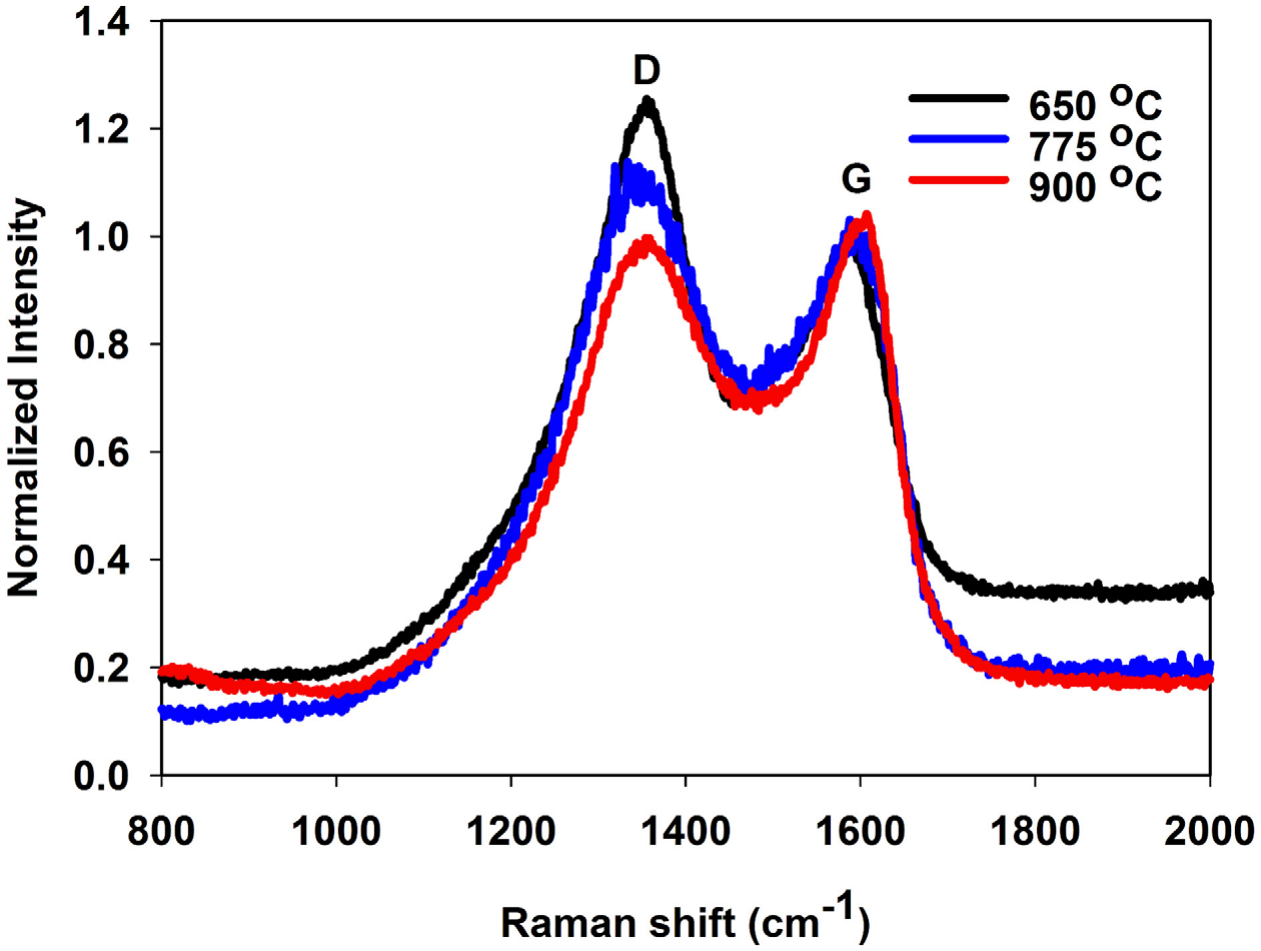
**Raman spectrum of sample A2 (TiO_2_@C), demonstrating the drop of D band vs. the G band as the graphitization temperature is increased**.

Raman spectroscopy was used to investigate the type of carbon allotropes formed through thermal treatment (Figure 5). The peaks D and G at around 1346 and 1590 cm^−1^, respectively correspond to sp^2^ carbon atoms in a disordered environment and to carbons in extended p conjugated graphite-like arrangements (Ferrari and Robertson, 2000; Ferrari, 2007) The ratio of the intensity of D to G peaks, called graphitization degree, is an indication of the amount of graphite carbon formed at the surface of the nanoparticle (lower values indicate greater amounts). The degree of graphitization (Table 2) decreases with graphitization temperature. As the temperature is raised, the thermal energy of the system becomes sufficient to convert structural defects into graphitic carbons. Interestingly, the graphitization degree obtained through our PAN thermal decomposition process at 775°C (0.85–0.96) is slightly lower than the one obtained via conventional hydrothermal treatment process at 800°C (1.00–1.18) (Zhang et al., 2008), an effect that we attribute to the preorganization of the extended carbon structure in the ladder -like carbonized PAN. The use of a PAN as template for the formation of the carbon layer is also advantageous as it allows tailoring the thickness of the carbon shell (Table 2).

### Photocatalytic activity of the TiO_2_@C nanoparticles

In the previous section, we have demonstrated that the encapsulation process is applicable for rutile, anatase and P25 (a composite of anatase and rutile). However, despite its greater band gap energy (3.2 vs. 3.0 eV) anatase is a better photocatalyst than rutile (Luttrell et al., 2014), and therefore we concentrated on anatase samples for the rest of this work. All further experiments were performed with samples graphitized at 650°C. The band gap energy of each sample, E_*g*_, was determined using Tauc's equation (Tauc et al., 1966; Zhang et al., 2011):

(1) $${\left(\alpha \text{h}\nu \right)}^{0.5}=\text{K}\left(\text{h}\nu -{\text{E}}_{\text{g}}\right)$$

where α is the absorption coefficient, K is a frequency independent constant, h is Planck's constant and ν is the frequency. The data were presented under the form of Tauc's plot (supplementary information), whereby (αhν)^0.5^ was plotted vs. hν, and the band gap energy was found at the intersect between the steep part of the curve and the x axis. The band gap energy of the TiO_2_@C samples (2.6–2.7 eV) is lower than the one of pure anatase (Table 2), indicating that the ability to absorb visible light is enhanced, and therefore TiO_2_@C are promising photocatalysts.

Electrochemical characterization was performed by preparing films of the TiO_2_@C nanoparticles deposited on transparent ITO glass. The preparation of the film was found to be of the crucial to insure reproducibility and stability of the electrical signals. The TiO_2_@C nanoparticles were dispersed in NMP containing a small amount of PVDF as binder. The sample was then spin-coated on ITO and dried. When immersed in water, the film was found to turn hazy and to blister, with a gradual loss of adhesion. Thus, ITO was first silanized prior spin-coating in order to ensure long term adhesion of the TiO_2_@C film. After silanization, no blistering and no hazing occurred, and the electrochemical measurements became reproducible.

**Figure 6:**
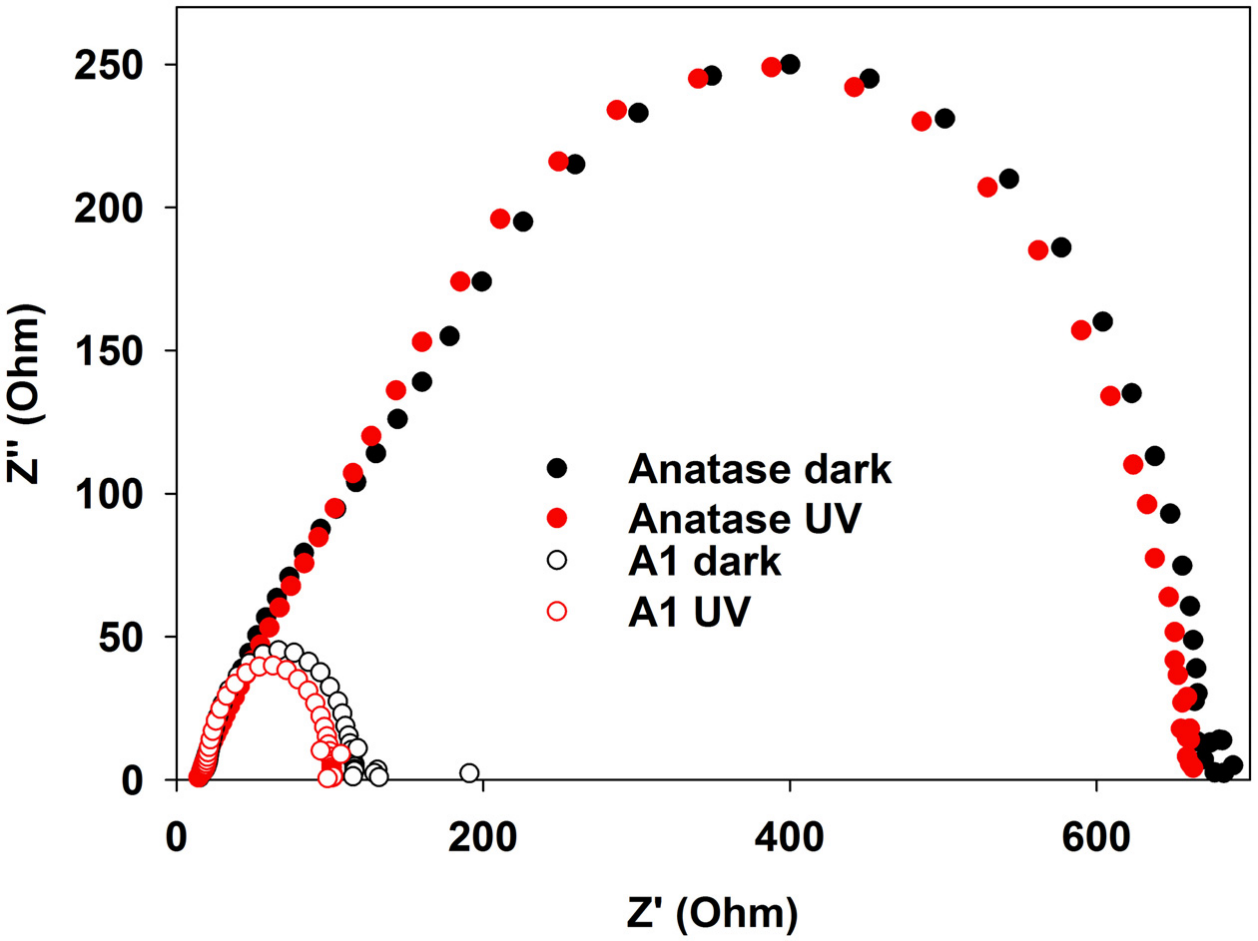
**Imaginary vs. real impedance (Nyquist graph) for anatase and TiO_2_@C (sample A1 graphitized at 650°C)**. UV (Xe lamp) illumination of nominal intensity 80 mW/cm^2^, measured intensity at surface: 3 mW/cm^2^.

Nyquist plots (Figure 6) clearly demonstrate the role played by the carbon layer. For TiO_2_, the impedance is large and it is not significantly affected by the presence of UV light. In stark contrast, the impedance of the TiO_2_@C sample is much lower, indicating that the conducting carbon layer facilitates the transport of the charge carriers through the system. Furthermore, in the presence of UV light, the impedance is further decreased, indicating that the carbon layer is also facilitating the separation of photogenerated carriers.

Cyclic voltammetry was used to assess the nature of the electrochemical events taking place in this system (several CV are shown in supplementary material section). The zero current potential, V_oc_, was found to be equal to 0.27 V. At higher imposed potentials, positive currents corresponding to oxidation of water are observed, and at an imposed potential 1.5 V, the oxidation becomes dominant. Thus, potentiostatic experiments were performed at an imposed potential of 1.5 V with alternated periods of darkness and light, in order assess the enhancement generated by the photocatalyzed generation of electron-holes pairs. Light was either produced by a UV source or by a solar simulator. In all cases, the presence of a carbon layer resulted in an enhancement of the photocurrent, enhancement which could be as high as 6 times greater. The response to UV is considerably greater than the response to solar illumination, as the band gap of anatase (3.2 eV) is more closely matched with the UV spectrum (Table 3).

**Table 3:** **Comparison of induced photocurrent under UV and solar illumination (samples pyrolyzed at 650°C)**.

**Sample**	**UV induced current (mA)**	**Solar light induced current (μA)**
Anatase	0.039	2.0
A1	0.130	1.4
A2	0.244	5.8

## Discussion

Several salient features of the encapsulation method presented here are worthy of being emphasized. First, the method is versatile, as it works equally well on anatase, rutile and P25 nanoparticles. Our method does only rely on a physical adsorption mechanism of a hydrophobic oligomer (PABA) in water, and it does not require covalent bonding between TiO_2_ surface sites and the polymer. As a result, this method is efficient irrespective of the nature of the TiO_2_ facet which is exposed at the surface. Furthermore, the absence of covalent bonding is noteworthy, as covalent bonds may significantly alter the electronic and optical properties of the TiO_2_, for example via the formation of a heterostructure. Importantly, the encapsulation method is not only applicable to TiO_2_ particles, but also to a variety of other inorganic species, as we (Das et al., 2011; Das and Claverie, 2012; Zhong et al., 2012) and others (Nguyen et al., 2008, 2013; Ali et al., 2009) have demonstrated in the past. Although we and others have worked so far in water, it should also be mentioned that we recently adapted this encapsulation technique for the encapsulation of nanotubes in organic solvents (Nguendia et al., 2014). Thus, this versatile nanoencapsulation method could be used to generate carbon shells on a great number of particles, and could become an interesting alternative to the hydrothermal treatment.

Another important feature of this nanoencapsulation technique is that due to the preorganization of the polymer around the polymer prior carbonization, the resulting carbon shell is highly uniform. The C shell thickness was measured on over 50 different locations in TEM pictures and was found to be relatively constant (the relative standard deviation is less than 35%, Table 2). In stark contrast, the usual hydrothermal synthesis leads to significantly more disperse shells, with standard deviations of 146% (resp 83, 79%) in the case of anatase (resp rutile, P25) nanoparticles (see supplementary information for relevant TEM pictures). Furthermore, conventional hydrothermal synthesis leads to shells which are often very thin. Although the average shell thickness is around 2–3 nm, most of the coating is in fact around 1 nm thick, but a few spots are much thicker (carbon deposits), resulting in an average thickness which is greater than 1 nm. Our nanoencapsulation method ensures that carbon is uniformly distributed, and therefore carbon deposits are absent. As a result, the thickness of the carbon layer can be very simply adjusted by solely adjusting the amount of AN vs. TiO_2_ in the emulsion polymerization recipe, as demonstrated for samples A1, A2, and A3. Preliminary results on the photocatalytic activity presented here indicate that carbon layer thickness indeed influences the amount of photocurrent (Table 3), and therefore the catalytic activity of the TiO_2_ particles. The results presented here establish a proof-of-concept that polymeric nanoencapsulation is a viable method to consistently construct uniform carbon layers with tunable thickness, and we intend to apply this method in the future to derive structure-activity relationships, relationships which are crucially lacking currently in literature, as, to our knowledge, no other method allows to efficiently control the thickness of the carbon layer.

### Conflict of interest statement

The authors declare that the research was conducted in the absence of any commercial or financial relationships that could be construed as a potential conflict of interest.
