# Supplementary material for: TiO2@C core-shell nanoparticles formed by polymeric nano-encapsulation
Source: Front Chem. 2014 Jul 11;2:47. doi: 10.3389/fchem.2014.00047 (PMC4092372; doi:10.3389/fchem.2014.00047)

**Supplementary material**

**TiO_2_@C Core-Shell Nanoparticles Formed by Polymeric Nano-Encapsulation**

**Mitra Vasei, Paramita Das, Hayet Cherfouth, Benoit Marsan, Jerome P. Claverie***

NanoQAM, Québec Center for Functional Materials, Dept of Chemistry, UQAM, Montreal, Qc, Canada

*** Correspondence:** Jerome Claverie, NanoQAM, Québec Center for Functional Materials, Dept of Chemistry, UQAM, Succ Centre-Ville, CP8888, Montreal, Qc, Canada

claverie.jerome@uqam.ca

**Figure S1** Absorption spectra of the TiO_2_ samples (graphitized at 650 ^o^C)

**Figure S2** Tauc plot of the TiO_2_@C samples graphitized at 650 ^o^C. The absorption value was corrected to account for the sample turbidity which was measured at 800 nm (at 800 nm, the measured absorption is due to elastic scattering and not to photon absorption). The values of the band gap are read at the intercept between the tangent line (thin line) and the x axis.

**Figure S3** Anatase nanoparticles before encapsulation


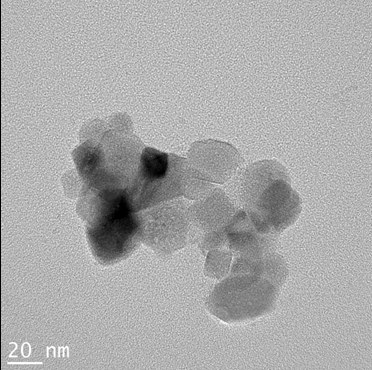


**Figure S4** Rutile snanoparticles before encapsulation


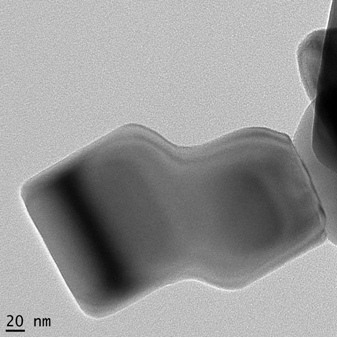


**Figure S5** P25 nanoparticles before encapsulation


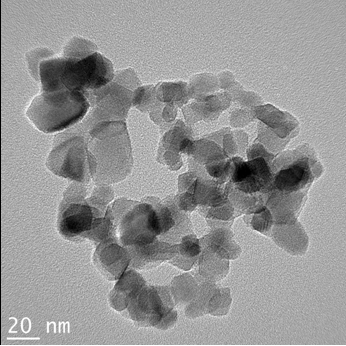


**Figure S6** TEM of sample R1 – TiO_2_@PAN and EDX analysis of one of the particle


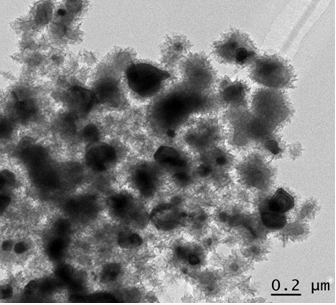

**Figure S7** TEM of sample R1 – TiO_2_@C (T = 650 ^o^C , EDX analysis of one of the particle and histogram of the C shell thickness.


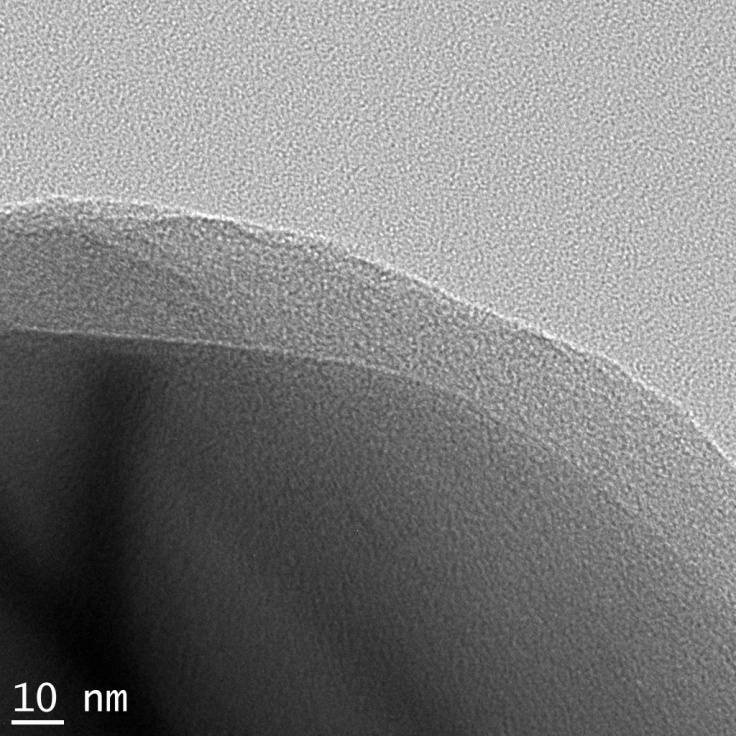

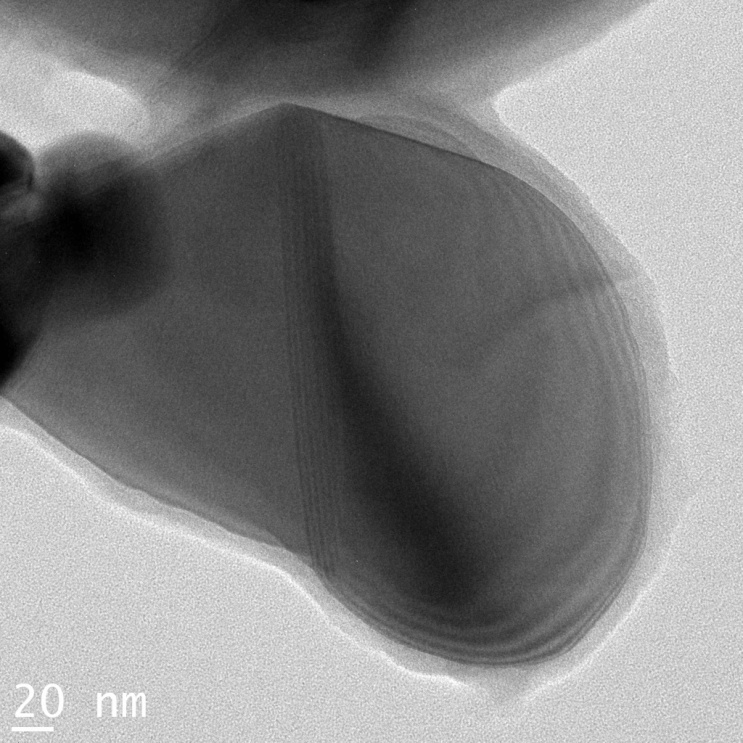

Figure S8 TEM of sample P1 – TiO_2_@PAN and EDX analysis of one of the particle


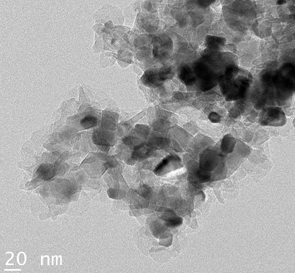

Figure S9 TEM of sample P1 – TiO_2_@C (T = 650 ^o^C), EDX analysis of one of the particle) and histogram of the C shell thickness.


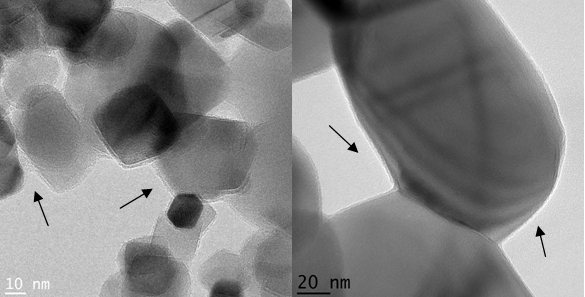

Figure S10 TEM of sample A1 – TiO_2_@PAN


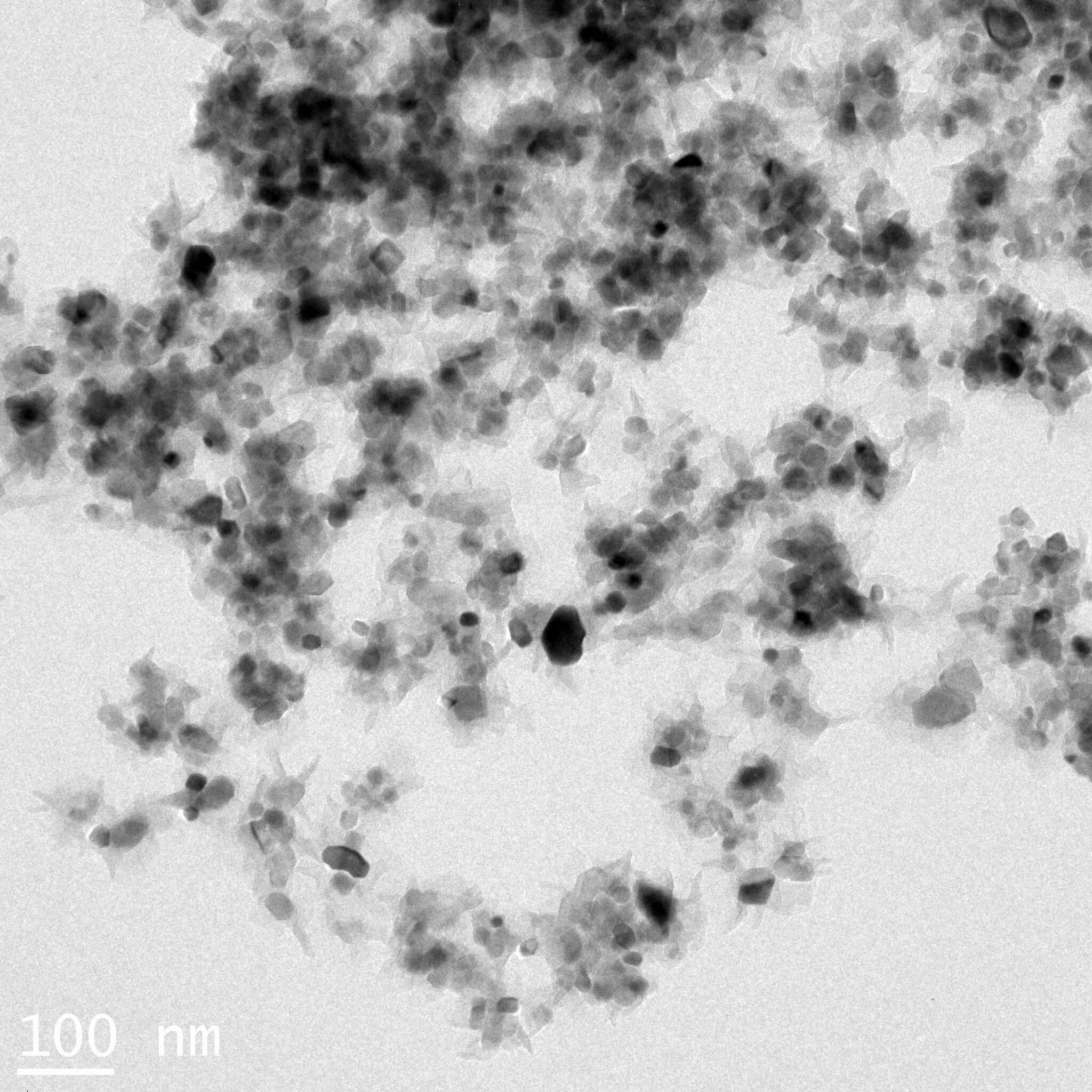


**Figure S11** TEM of sample A1 – TiO_2_@C (3 pictures) – Sample pyrrolized at 775 ^o^C and shell thickness histogram


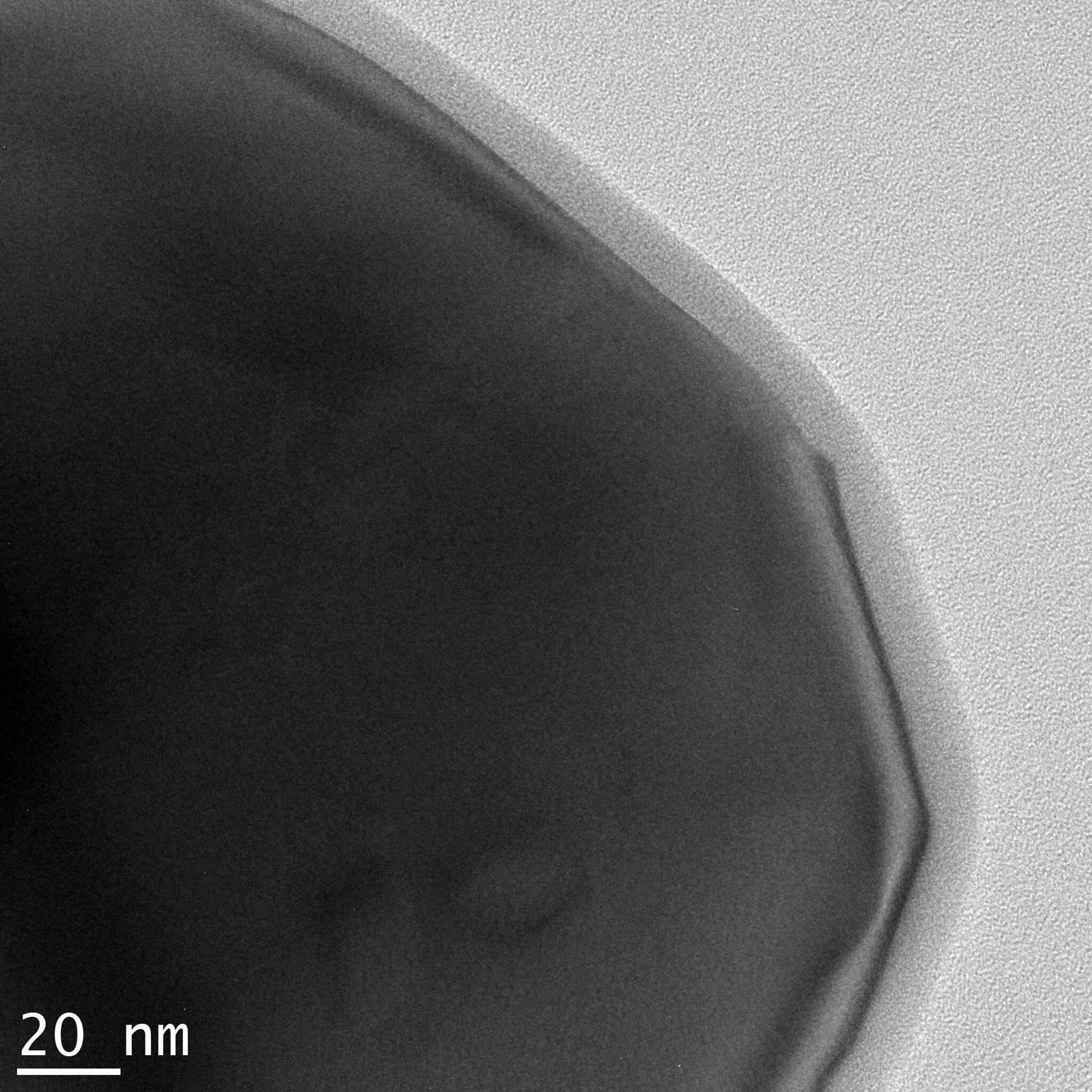

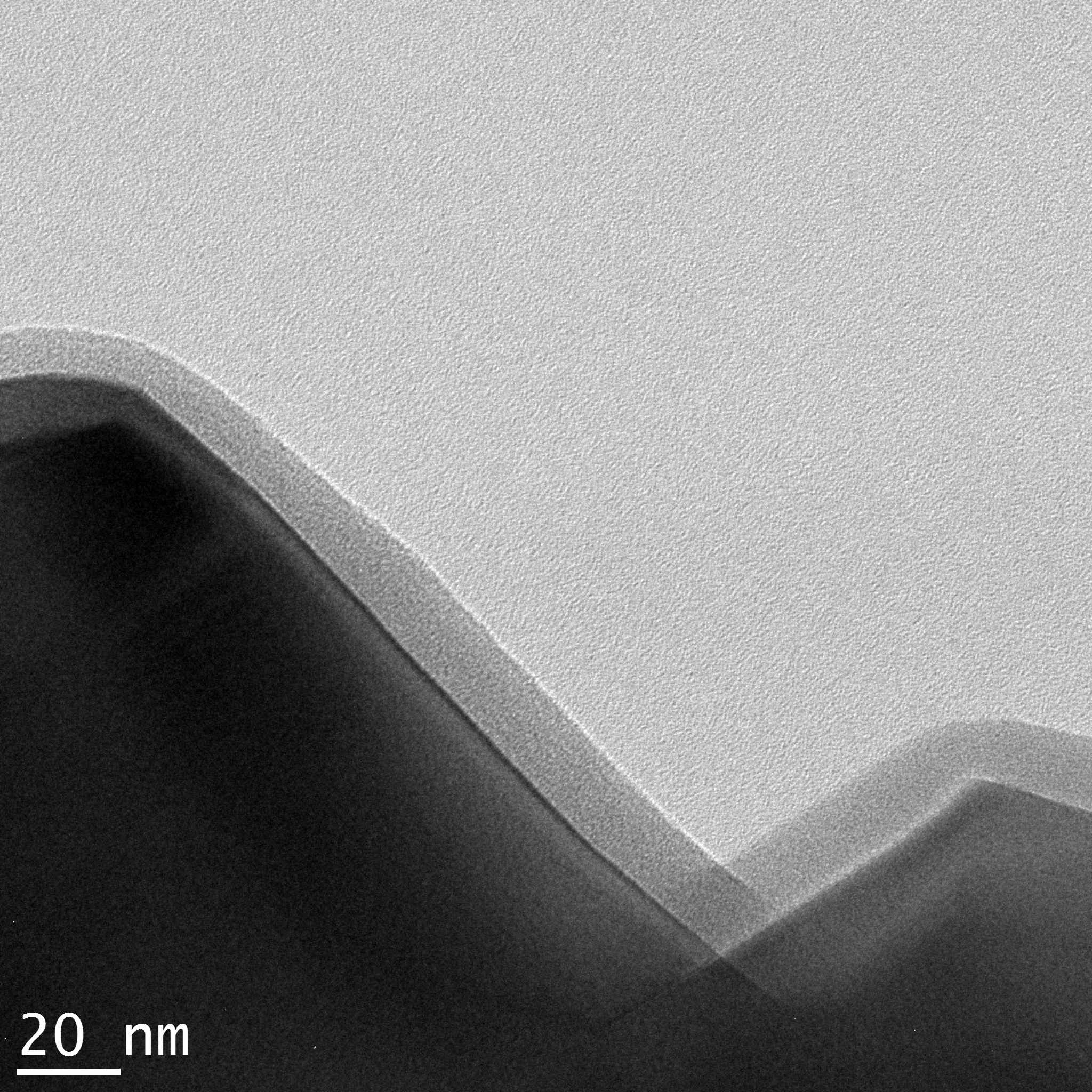

**Figure S12** Sample A1 pyrolyzed at 900 ^o^C (notice the formation of rodlike rutile particles).


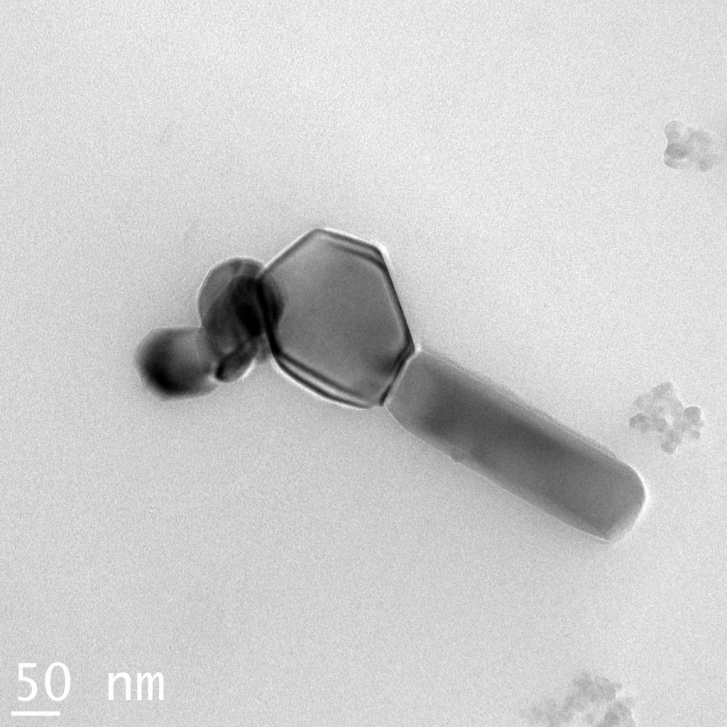


**Figure S13** rutile@C prepared using conventional hydrothermal treatment and shell thickness histogram


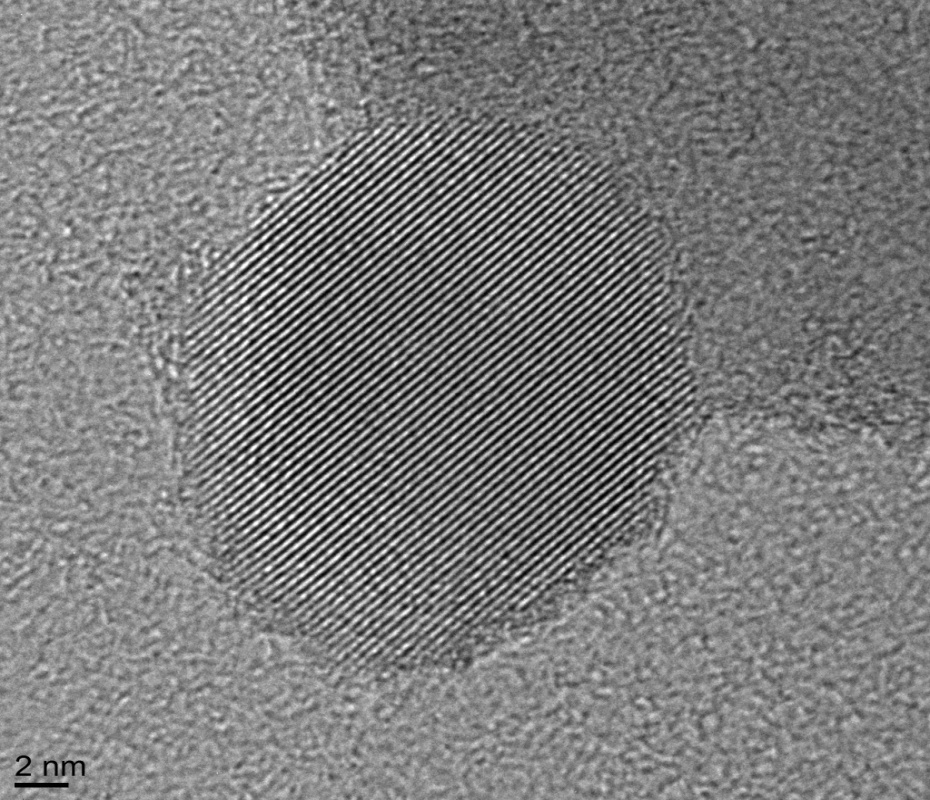

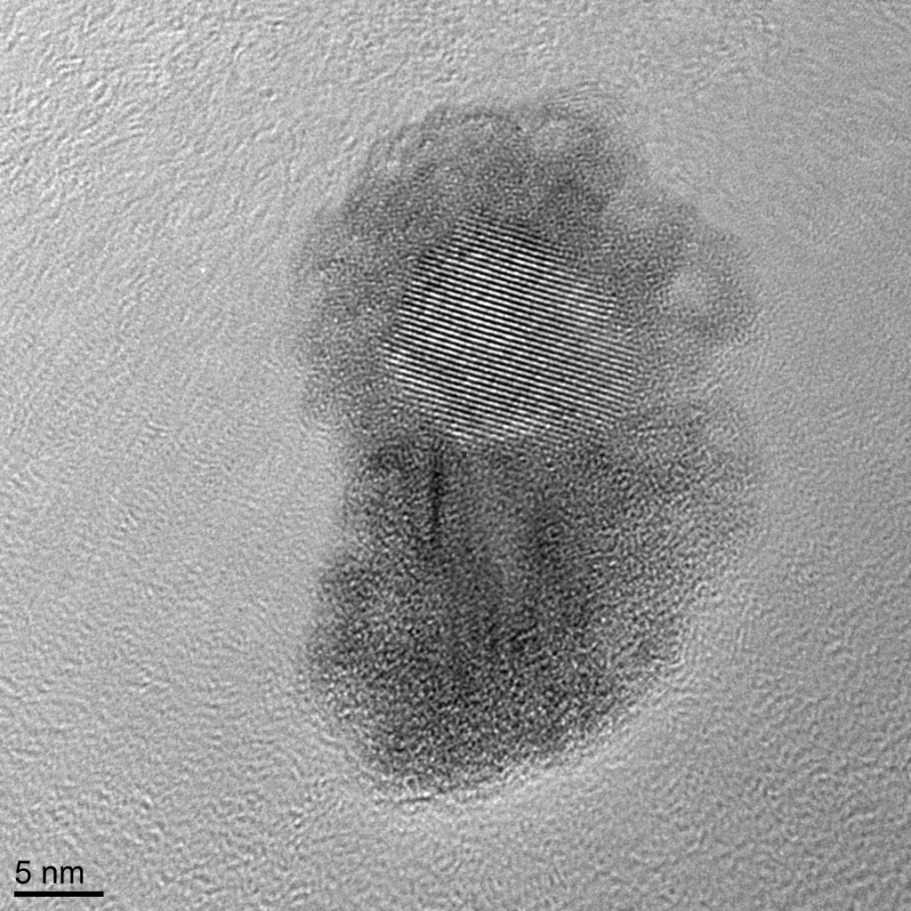

**Figure S14** P25@C prepared using conventional hydrothermal treatment and shell thickness histogram


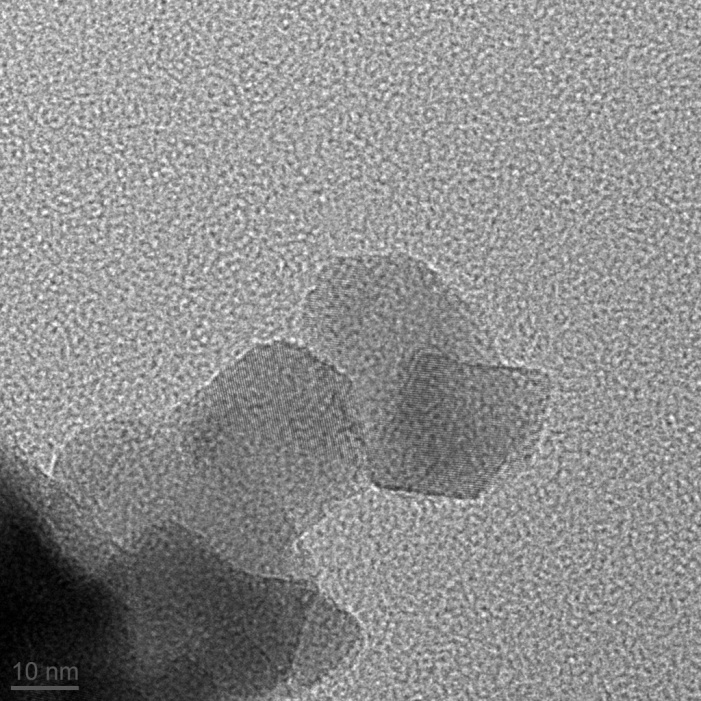

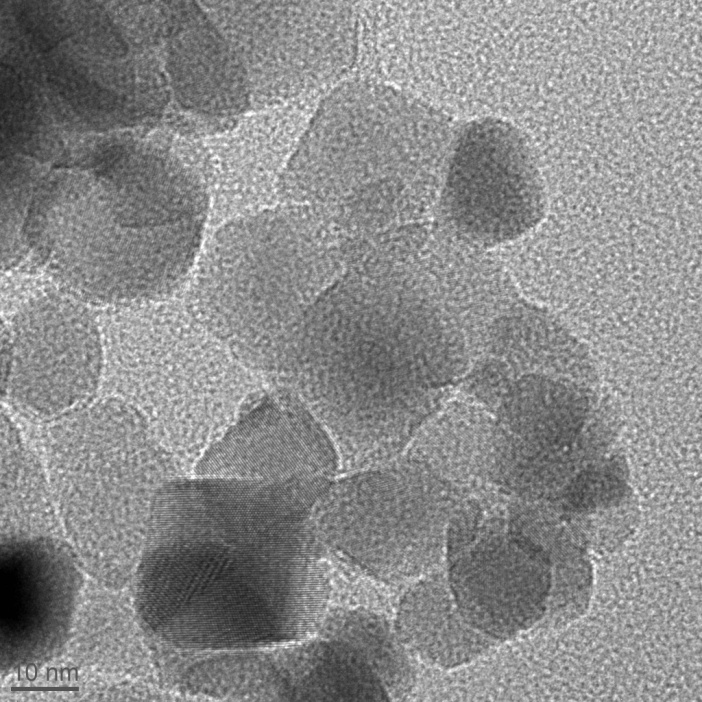

**Figure S15** anatase@C prepared using conventional hydrothermal treatment and shell thickness histogram


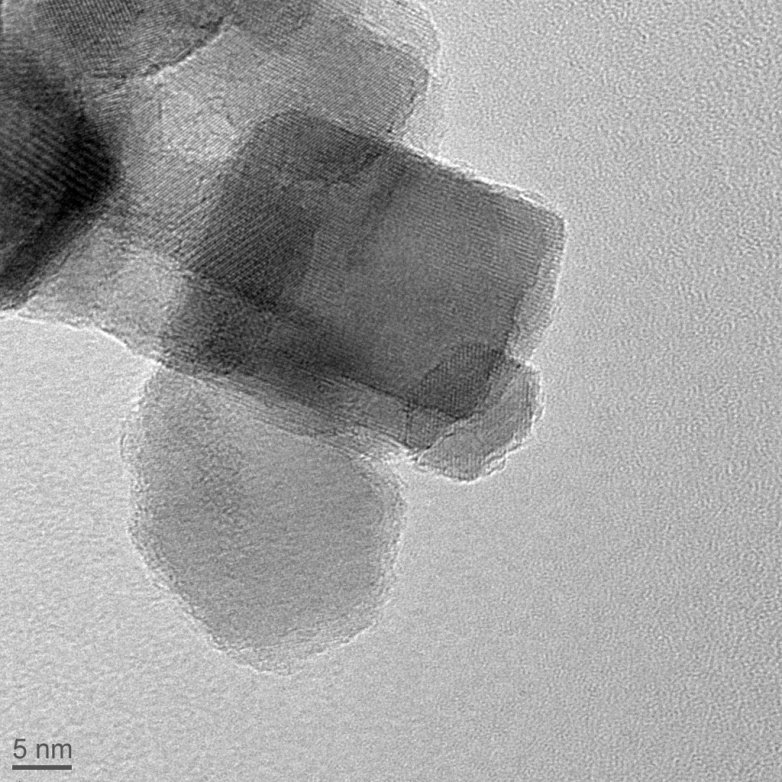

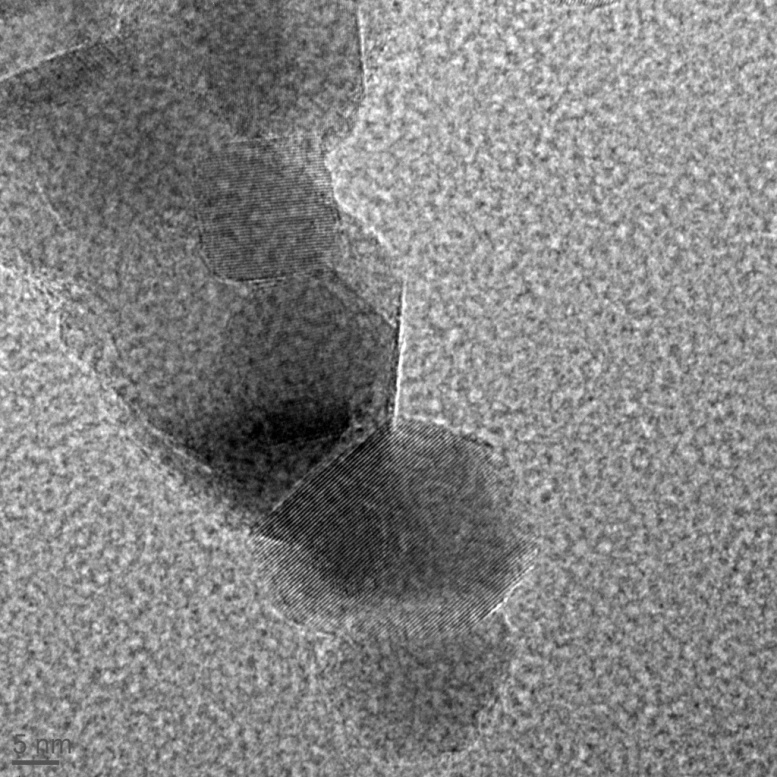

**Figure S16** CV of the anatase samples pyrolyzed at 650 °C (0.5 to 1.5 V – scan rate = 100 mV/s, Na_2_SO_4_/H_2_SO_4_,Ct:Pt,Ref:Ag/AgCl,pH=2.5, Surface=1 cm^2^ )

**Figure S17** Nyquist plot for A2 sample (UV (Xe lamp) illumination 80 mW/cm^2^, Intensity at surface 3 mW/cm^2^ E=1.5V,Na_2_SO_4_/H_2_SO_4_,Ct:Pt,Ref:Ag/AgCl,pH=2.5 Surface=1 cm^2^)

**Figure S18** Potentiostatic measurements (Solar simulator, illumination 80 mW/cm^2^, intensity at surface 3.7 mW/cm^2^, E=1.5V,Na_2_SO_4_/H_2_SO_4_,Ct:Pt,Ref:Ag/AgCl,pH=2.5, surface=1 cm^2^ )

**Figure S19** Potentiostatic measurements. The two graphs could not be put on the same scale due to the large current for sample A2 and low current for pure anatase. (UV (Xe lamp), illumination 80 mW/cm^2^, intensity at surface 3 mW/cm^2^, E=1.5V, Na_2_SO_4_/H_2_SO_4_, Ct:Pt, Ref:Ag/AgCl, pH=2.5, surface=1 cm^2^ )


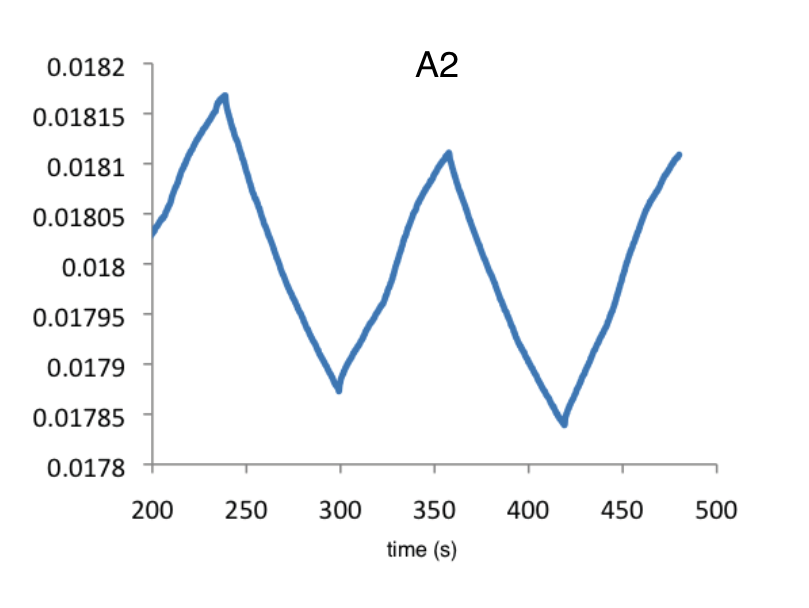

Supplement: Supplementary file 1 [file DataSheet1.DOCX]
